# Clinical characteristics and management of headache in patients with myeloproliferative neoplasms

**DOI:** 10.3389/fneur.2022.1051093

**Published:** 2022-12-06

**Authors:** Takashi Shimoyama, Hiroki Yamaguchi, Kazumi Kimura, Fumiaki Suzuki, Toshiyuki Hayashi, Satoshi Wakita

**Affiliations:** ^1^Department of Neurology, Nippon Medical School, Tokyo, Japan; ^2^Department of Hematology, Nippon Medical School, Tokyo, Japan

**Keywords:** secondary headache disorder, MRI, myeloproliferative neoplasms (MPN), management—healthcare, low-dose aspirin

## Abstract

**Background:**

Headache is frequently reported as a neurological manifestation of myeloproliferative neoplasms (MPNs), including polycythemia vera and essential thrombocythaemia. This study sought to clarify the clinical characteristics and response to treatment of headaches in patients with MPNs.

**Methods:**

We prospectively studied 137 patients with MPNs. The following information was gathered to assess the features of headache at baseline and at follow-up (>6 months): (1) average duration of headache attacks, (2) number of headache days per month, (3) numerical rating scale (NRS), (4) Headache Impact Test-6 (HIT-6), and (5) Migraine Disability Assessment (MIDAS). We compared those parameters for headaches between the baseline and follow-up interviews according to the management.

**Results:**

Thirty-seven (27.0%) patients had headache. The prevalence of headaches gradually decreased with increasing age (Age ≤ 49 years: 61.0%, 50–59 years: 38.5%, 60–69 years: 17.2%, 70–79 years: 5.1%, and ≥80 years: 0.0%, *P* < 0.001). Multiple logistic regression analysis showed that younger age, but not platelet counts or the *JAK2* V617F mutation, was independently associated with headaches (Odds Ratios 2.004, 95% confidence intervals 1.293–3.108, *P* = 0.002). Scintillating scotomas were present in 22 (59.5%) of 37 patients with headaches, while four patients developed sudden headaches that lasted for only 0–10 min. Follow-up interviews were available for 31 (83.8%) of 37 patients with headaches. Twenty-one (67.7%) patients were treated with low-dose aspirin (100 mg once daily) [low-dose aspirin alone: *n* = 9; combined cytoreductive therapy: *n* = 12] for headache management. All parameters for headache [average duration of headache attacks, number of headache days per month, NRS score, HIT-6 score, and MIDAS score (all *P* < 0.001)] were significantly improved at follow-up in patients taking low-dose aspirin. However, there were no significant differences in these parameters of headaches in patients who did not receive low-dose aspirin.

**Conclusion:**

Headaches is common in patients with MPNs, particularly in younger patients. MPN-related headaches may be managed by using low-dose aspirin and controlling MPNs.

## Introduction

Migraine is a common and often disabling neurological disease that is typically characterized by recurrent attacks of moderate-to-severe, unilateral, pulsating headaches associated with nausea, vomiting, photophobia, and phonophobia (1). The 2016 Global Burden of Disease data estimated that the prevalence of migraine was 14.4% overall, 18.9% in women, and 9.8% in men in the general population (2). Several disorders are associated with migraine features, including stroke (3), patent foramen ovale (PFO) (4), epilepsy (5), psychiatric disorders (6), and sleep disorder (7). The International Committee of Headache Disorders, 3rd edition (ICHD-3) classifies a headache or a headache disorder that is secondary to another disorder as secondary headaches attributed to those disorders (1). Understanding specific disorders that present with headaches is important for the investigation of additional diagnostic approaches [e.g., transesophageal echogram for PFO, electroencephalogram for epilepsy, and genetic analysis for cerebral autosomal dominant arteriopathy with subcortical infarcts and leukoencephalopathy (8)].

Myeloproliferative neoplasms (MPNs), including polycythemia vera (PV) and essential thrombocythemia (ET), are chronic clonal proliferations of myeloid cells characterized by erythrocytosis and thrombocytosis (9, 10). The valine 617 phenylalanine (V617F) mutation in the gene encoding Janus kinase 2 (*JAK2*) is detected in ~95% of patients with PV and 50–70% of patients with ET (11–13). In general, thromboembolic events, including stroke, myocardial infarction, and deep vein thrombosis, have been considered as major complications of MPNs in previous reports (13–15). Interestingly, ~30% of patients with MPNs develop vasomotor symptoms that present with transient neurological manifestation, including headache, dizziness, numbness, and visual disturbance (16, 17). Michiels et al. (18) reported transient ischemic neurological or visual symptoms in 17 patients with ET, 10 of whom presented with pulsating headaches. All attacks had a sudden onset, lasted for a few seconds to several minutes, and were usually associated with a dull pulsatile headache (18). However, differential diagnosis from migraine *per se* may be difficult, as some MPN-related headaches present with symptoms strikingly similar to those of migraine with typical aura (19, 20).

However, the management of MPN-related headaches seems to differ from the treatment strategy for migraine. Pharmacological management of migraine includes acute and preventive treatment of attacks (21). Non-steroidal anti-inflammatory drugs (NSAIDs) or triptans are commonly used for acute migraine treatment (21). Preventive agents include β-blockers, antiepileptics, antidepressants, and calcitonin gene-related peptide (CGRP) monoclonal antibodies (21). In patients with MPN-related headaches, several reports have suggested the efficacy of low-dose aspirin and/or reduction of platelet count using cytoreductive therapy (18–20, 22–24). However, to date, previous studies have not investigated whether aspirin and/or cytoreductive therapy influences the number of headache days per month, or scores on scales, such as the numerical rating scale (NRS), headache impact test-6 (HIT-6), and migraine disability assessment (MIDAS), which are often used to evaluate the efficacy of treatments for episodic or chronic migraine (25). The present study aimed to clarify the clinical characteristics and response to treatment of headaches in patients with MPNs.

## Methods

### Patients

We prospectively enrolled patients with MPNs who were treated at Nippon Medical School Hospital between September 2017 and March 2022. MPNs were diagnosed if they met the 2016 World Health Organization diagnostic criteria for MPNs (9). All participants underwent genetic mutation analysis of *JAK2 V617F, CALR*, and *MPL* genes. We evaluated brain magnetic resonance imaging (MRI) abnormalities in patients with MPNs, unless there were contraindications to MRI (e.g., cardiac pacemakers) or patient refusal. Detailed genetic mutation analysis methods, brain MRI protocols, and assessments of brain MRI abnormalities have been reported previously (26). Management of MPNs, including the use of anti-thrombotic agents, cytoreduction therapy, and phlebotomy, was conducted by hematology and neurology specialists based on the current treatment recommendations and risk stratification (13). This observational study was approved by the institutional ethics committee of the Nippon Medical School Hospital. Written informed consent was obtained from all participants.

### Baseline clinical characteristics

The following clinical data were recorded for all patients at baseline: (1) age and sex; (2) vascular risk factors, including hypertension, diabetes mellitus, and dyslipidemia; (3) atrial fibrillation (AF); (4) current smoking and alcohol status; (5) medical history of thrombotic events (ischemic stroke and ischemic heart disease); (6) prior history of headache disorder (tension-type and migraine) before the diagnosis of MPNs, (7) brain MRI findings; (8) gene mutation profiles; (9) laboratory parameters at MPN diagnosis, namely white blood cell count (WBC), hemoglobin (Hb), and platelet count; and (10) treatments for MPN [low-dose aspirin (100 mg once daily) and cytoreductive therapy].

### Vasomotor symptoms and headaches

We assessed the presence of vasomotor symptoms, including headache, vertigo/dizziness, tinnitus, scintillating scotomas, diplopia, blurred vision, and acroparesthesia/numbness. The presence of headache was defined by thev fulfillment of at least two of the following four characteristics for migraine without aura according to ICHD-3 criteria (1): (A) unilateral location, (B) pulsating quality, (C) moderate or severe pain intensity, and (D) aggravation by or avoidance of routine physical activity. In patients with headaches, we obtained the following information: (1) average duration of headache attacks: (i) 0–10 min, (ii) 10–60 min, (iii) 1–2 h, (iv) 2–4 h, (v) 4–72 h, (2) number of headache days per month, (3) whether scintillating scotomas were present, (4) the NRS score during the headache attack, (5) the HIT-6 score, and (6) the MIDAS score. Headache data (e.g., occurrence, duration, and pain intensity) were collected through participant recall. Management of headache and MPNs [e.g., NSAIDs, triptans, migraine-preventive agents, low-dose aspirin (100 mg once daily), and cytoreductive therapy] during the follow-up were based on the attending physician's judgment. A face-to-face follow-up interview was performed at 6 months after the initial interview.

### Statistical analysis

First, patients with MPNs were assigned to one of two groups based on the presence or absence of headaches. Baseline clinical characteristics were compared between the two groups. Next, a multiple logistic regression analysis was conducted to identify the independent factors associated with headaches in patients with MPNs. The variables from univariate analyses with *P*-values < 0.1 were entered into this analysis. Odds ratios (ORs) are presented with 95% confidence intervals (CIs). Finally, we compared average duration of headache attacks, number of headache days per month, and NRS, HIT-6, and MIDAS scores between the baseline and follow-up interviews. Patients with missing follow-up interviews were excluded from this analysis. Continuous variables are expressed as a median and interquartile range in the text and tables. The significance of intergroup differences was assessed using the χ^2^-test for categorical variables and the Mann–Whitney *U*-test for continuous variables in the univariate analysis. Statistical significance was set at *P* < 0.05. All statistical analyses were performed using Statistical Package for the Social Sciences software for Windows (SPSS version 26.0, Chicago, IL, USA).

## Results

### Baseline clinical characteristics and incidence of vasomotor symptoms

**Figure 1:**
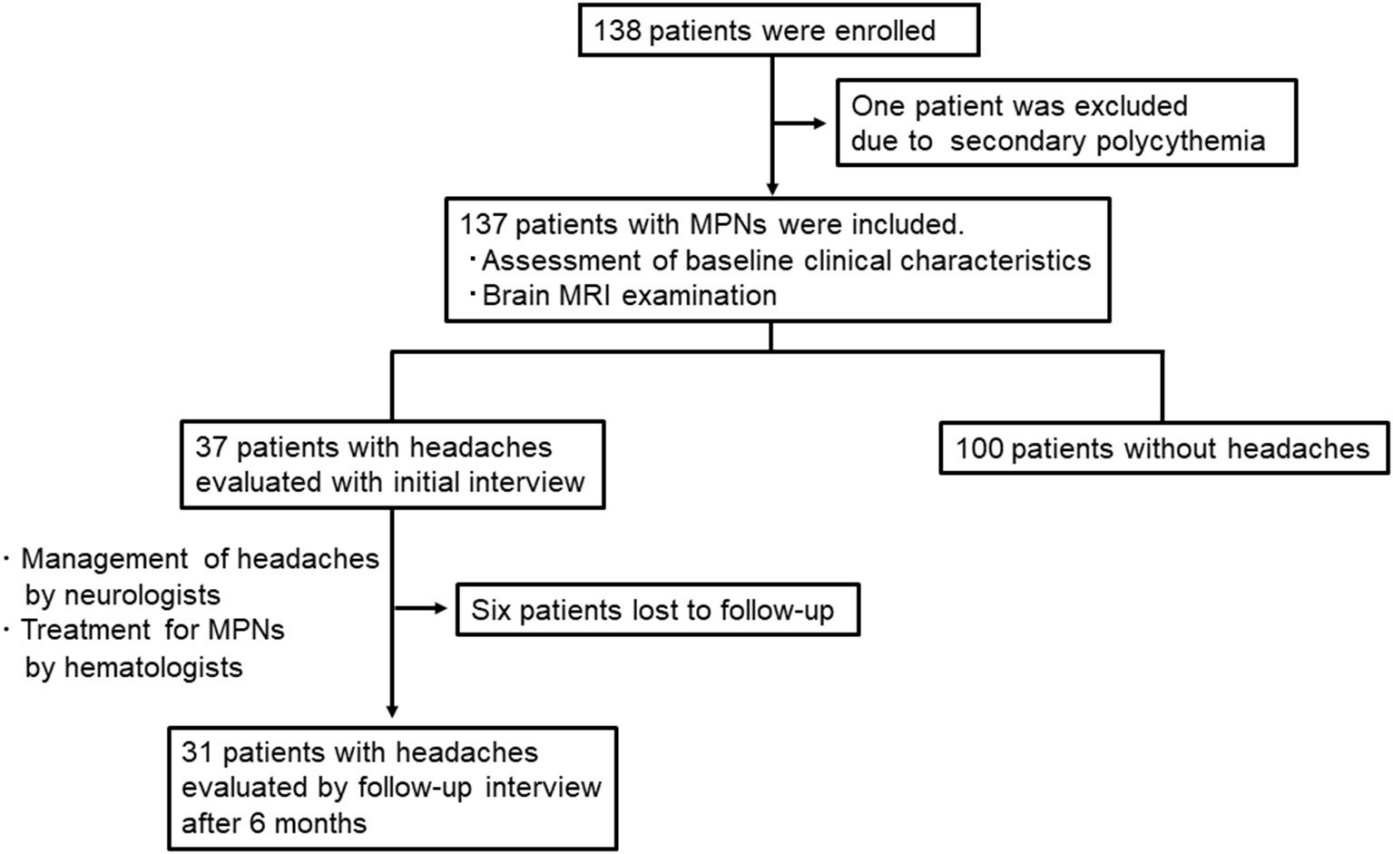
Time frame and study flow gram in this study.

A total of 138 patients were enrolled in the study. Of these, we excluded one patient who was diagnosed with secondary polycythemia. Finally, 137 patients with MPNs [75 women; median age 67 (47–75) years; PV: 45 and ET: 92] were analyzed in the present study (Table 1). The time frame and study flow gram were presented in Figure 1. Thirteen patients (9.2%) had a prior history of tension-type headache, while 12 patients (8.8%) were previously diagnosed with migraine before the diagnosis of MPNs. The *JAK2V617F* mutation was detected in 70.2% of MPN patients. At the baseline investigation, some form of vasomotor symptom was observed in 69 patients (50.4%). Headache (27.0%) was the most common manifestation in MPN patients, followed by vertigo/dizziness (22.6%), scintillating scotomas (16.8%), acroparesthesia/numbness (12.4%), tinnitus (10.2%), blurred vision (4.4%), and diplopia (1.5%). In this study, three patients refused brain MRI after having given written informed consent for study participation, and one patient could not undergo MRI due to a pacemaker. Thus, brain MR images were available for 133 (97.1%) of 137 patients with MPNs.

**Table 1:** Baseline demographics in patients with MPNs.

	***n* = 137**
Age, y, median (IQR)	67 (47–75)
Gender, Female, *n* (%)	75 (54.7)
ET, *n* (%)	92 (67.2)
Stroke risk factor, *n* (%)	
Hypertension	68 (49.6)
Dyslipidemia	28 (20.4)
Diabetes mellitus	15 (10.9)
Atrial fibrillation	7 (5.1)
Smoking	44 (32.1)
Alcohol	59 (43.1)
History of thrombosis, *n* (%)	
Ischemic stroke	21 (15.3)
Ischemic heart disease	8 (5.8)
History of headache, *n* (%)	
Tension-type	13 (9.2)
Migraine	12 (8.8)
Genetic Mutations, *n* (%)	
JAK2V617F	96 (70.1)
CALR	6 (4.4)
MPLW515L/K	2 (1.5)
No mutations	33 (24.1)
Laboratory findings	
WBC (×10^2^/mm^3^) (IQR) at diagnosis	95 (71–128)
Hemoglobin (g/dL) (IQR) at diagnosis	14.1 (13.0–16.4)
Platelets (×10^4^ mm^3^) (IQR) at diagnosis	66.5 (50.1–94.3)
Any vasomotor symptoms	69 (50.4)
Headaches	37 (27.0)
Vertigo/dizziness	31 (22.6)
Tinnitus	14 (10.2)
Scintillating scotomas	23 (16.8)
Diplopia	2 (1.5)
Blurred vision	6 (4.4)
Acroparesthesia/numbness	17 (12.4)
Treatments for MPNs at baseline	
Low-dose aspirin	82 (59.9)
Cytoreductive therapy	67 (48.9)
MRI findings^*^, *n* (%)	
Brain infarcts	31/133 (23.3)
WMLs	41/133 (30.8)
CMBs	12/133 (9.0)
Large vessel involvement	12/133 (9.0)

MPNs, myeloproliferative neoplasms; IQR, interquartile; ET, essential thrombocythemia; WBC, white blood cell; MRI, magnetic resonance imaging; WMLs, white matter lesions; CMBs, cerebral microbleeds.

* Four patients did not evaluate brain MRI.

### Factors associated with headaches

**Figure 2:**
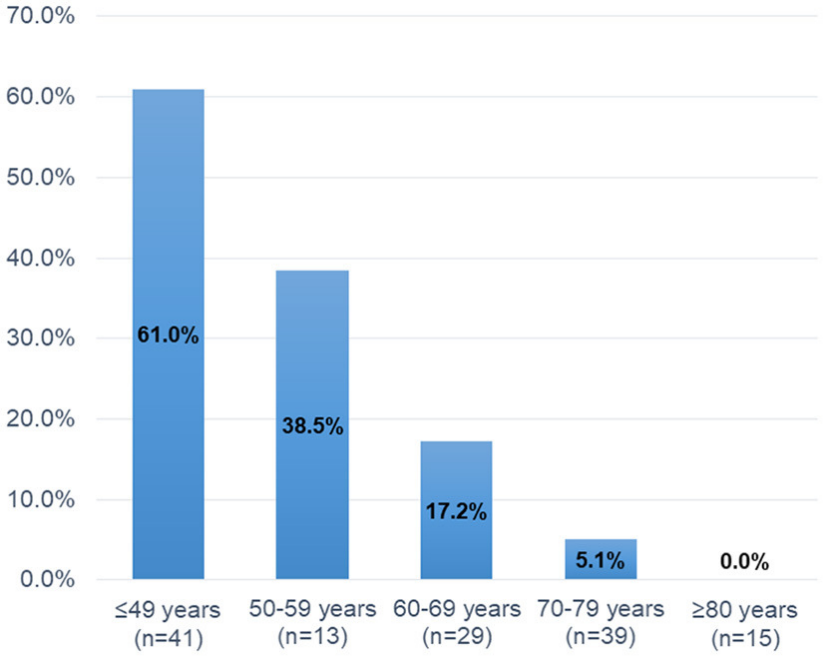
Prevalence of headaches according to age category.

Table 2 shows the clinical characteristics of MPN patients with or without headaches. Patients with headaches were more likely to be younger (45 vs. 70 years, *P* < 0.001), female (70.3 vs. 49.0%, *P* = 0.033), and have ET (89.2 vs. 59.0%, *P* < 0.001) than were patients without headaches. The prevalence of headaches gradually decreased with increasing age (Age ≤ 49 years: 61.0%, 50–59 years: 38.5%, 60–69 years: 17.2%, 70–79 years: 5.1%, and ≥80 years: 0.0%, *P* < 0.001; Figure 2). Hypertension was less common in patients with headaches than in those without headaches (18.9 vs. 61.0%, *P* < 0.001). The presence of the *JAK2V617F* mutation (70.3 vs. 70.0%, *P* > 0.999) and laboratory parameters (WBC, *P* = 0.080; Hb, *P* = 0.860; platelets: *P* = 0.141) were not significantly different between those with and those without headaches. Brain MRI scanning was available for all patients with headaches, whereas four patients without headaches did not undergo brain MRI scanning. Brain MRI abnormalities, including brain infarcts [10.8% (4/37) vs. 28.1% (27/96), *P* = 0.040] and white matter lesions [WMLs; 13.5% (5/37) vs. 37.5% (36/96), *P* = 0.011] were less frequently observed in patients with headaches than in those without headaches. Treatment for MPNs at baseline was less often administered for patients with headaches than for patients without headaches (low-dose aspirin: 24.3 vs. 62.0%, *P* < 0.001; cytoreductive therapy: 27.0 vs. 57.0%, *P* = 0.002), because treatments for MPNs are usually initiated in patients with a higher risk of thromboembolic events (age > 60 years, history of thrombosis and/or vascular risk factors, and presence of the *JAK2 V617F* mutation).

**Table 2:** Comparison of patients with headaches and without headaches.

	**Headaches (*n* = 37)**	**No headaches (*n* = 100)**	***P***
Age, y, median (IQR)	45 (37–55)	70 (61–77)	<0.001
Gender, Female, *n* (%)	26 (70.3)	49 (49.0)	0.033
ET, *n* (%)	33 (89.2)	59 (59.0)	<0.001
Stroke risk factor, *n* (%)			
Hypertension	7 (18.9)	61 (61.0)	<0.001
Dyslipidemia	5 (13.5)	23 (23.0)	0.339
Diabetes mellitus	1 (2.7)	14 (14.0)	0.069
Atrial fibrillation	0 (0.0)	7 (7.0)	0.189
Smoking	8 (21.6)	36 (36.0)	0.149
Alcohol	14 (37.8)	45 (45.0)	0.561
History of thrombosis, *n* (%)			
Ischemic stroke	4 (10.8)	17 (17.0)	0.436
Ischemic heart disease	1 (2.7)	7 (7.0)	0.682
Genetic Mutations, *n* (%)			
JAK2V617F	26 (70.3)	70 (70.0)	1.000
CALR	0 (0.0)	2 (2.0)	1.000
MPLW515L/K	2 (5.4)	4 (4.0)	0.661
No mutations	9 (24.3)	24 (24.0)	1.000
Laboratory findings			
WBC (×10^2^/mm^3^) (IQR) at diagnosis	87 (70–110)	100 (72–139)	0.080
Hb (g/dL) (IQR) at diagnosis	14.0 (13.6–15.3)	14.4 (12.6–17.1)	0.860
Plt (×10^4^ mm^3^) (IQR) at diagnosis	71.5 (54.4–92.5)	64.4 (46.7–93.7)	0.141
Treatments for MPNs at baseline, *n* (%)			
Low-dose aspirin	9 (24.3)	62 (62.0)	<0.001
Cytoreductive therapy	10 (27.0)	57 (57.0)	0.002
MRI findings^*^, *n* (%)			
Brain infarcts	4/37 (10.8)	26/96 (28.1)	0.040
WMLs	5/37 (13.5)	36/96 (37.5)	0.007
CMBs	1/37 (2.7)	11/96 (11.5)	0.178
Large vessel involvement	2/37 (5.4)	10/96 (10.4)	0.509

MPNs, myeloproliferative neoplasms; IQR, interquartile; ET, essential thrombocythemia; WBC, white blood cell; Hb, hemoglobin; Plt, platelets; MRI, magnetic resonance imaging; WMLs, white matter lesions; CMBs, cerebral microbleeds.

* Four patients did not evaluate brain MRI.

Table 3 shows the results of multiple logistic regression analyses for headaches. Factors with values of *P* < 0.10 in univariate analyses were age, female sex, ET, absence of hypertension and diabetes mellitus, and no treatments for MPNs (low-dose aspirin and cytoreduction therapy). In model 1 (all patients: *n* = 137), we adjusted for the *JAK2 V617F* mutation and laboratory parameters (WBC, Hb, and Plt). In model 2 (patients who underwent MRI; *n* = 133), we adjusted for model 1 and abnormal MRI findings (brain infarcts and white matter lesions). Multivariate logistic regression analysis showed that age (decrease per 10 years) was the only independent factor associated with headache (Model 1: ORs 2.004, 95% CIs 1.293–3.108, *P* = 0.002; Model 2: ORs 2.161, 95% CIs 1.364–3.422, *P* = 0.001). The other variables were not associated with the incidence of headaches in multivariate logistic regression analysis.

**Table 3:** Multivariate logistic analysis for headaches.

	**Model 1 (*****n*** = **137)**			**Model 2 (*****n*** = 133)^*^		
	**ORs**	**95% CIs**	***P***	**ORs**	**95% CIs**	***P***
Age per 10 years decrease	2.004	1.293–3.108	0.002	2.161	1.364–3.422	0.001
Female	2.243	0.713–7.060	0.167	2.634	0.785–8.833	0.117
ET	5.625	0.840–37.681	0.075	5.300	0.767–36.615	0.091
Hypertension	0.344	0.099–1.198	0.094	0.291	0.079–1.070	0.063
Diabetes mellitus	0.595	0.058–6.069	0.661	0.495	0.046–5.364	0.563
JAK2V617F	3.467	0.924–13.016	0.065	3.878	0.968–15.534	0.056
WBC	0.999	0.985–1.013	0.863	0.999	0.985–1.013	0.853
Hb	1.178	0.827–1.679	0.365	1.166	0.803–1.693	0.419
Plt	1.003	0.985–1.020	0.774	1.002	0.985–1.020	0.823
Low–dose aspirin	0.662	0.183–2.392	0.529	0.713	0.177–2.872	0.634
Cytoreductive therapy	1.334	0.321–5.534	0.692	1.131	0.270–4.729	0.866
Brain infarcts	-	-	-	0.396	0.068–2.292	0.301
WMLs	-	-	-	2.213	0.455–10.767	0.325

ET, essential thrombocythemia; WBC, white blood cell; Hb, hemoglobin; Plt, platelets; WMLs, white matter lesions; ORs, odds ratios; CIs, confidential intervals.

* Patients who underwent brain magnetic resonance imaging (MRI).

### Clinical characteristics of headaches at baseline

Table 4 shows the detailed baseline characteristics of headaches (*n* = 37). The features of headache were as followed; (A) unilateral location (*n* = 23, 62.2%), (B) pulsating quality (*n* = 12, 32.4%), (C) moderate or severe pain intensity (*n* = 27, 72.9%), and (D) aggravation by or avoidance of routine physical activity (*n* = 30, 81.1%). Scintillating scotomas were present in 22 patients (59.5%). The median average duration of headache attack was 3 (2–8) h, and in 22 patients (59.5%), it lasted <4 h [0–10 min: four (10.8%), 10–60 min: three (8.1%), 1–2 h: seven (18.9%), and 2–4 h: eight (21.6%) patients]. The median number of headache days per month was 4 (2–9), and 28 patients (75.6%) had <7 headache days in a month. The median headache intensity, evaluated using the NRS score, was 6 (5–8). Mild pain intensity (NRS ≤ 5) was observed in 10 patients (27.0%), whereas moderate (6–7) and severe (8–10) intensity was observed in 17 (45.9%) and 10 (27.0%) patients, respectively. The impact of headaches on life was assessed using the HIT-6 scale, and a median score of 52 (44–63) was found. Severe impact (HIT-6 score ≥ 60) on life was present in 13 patients (35.1%), while substantial, some, and little-to-no impact was present in one (2.7%), five (13.5%), and 18 (48.6%) patients, respectively. Headache severity and disability were assessed using the MIDAS, which yielded a median score of 3 (0–5). Severe disability (MIDAS score ≥ 21) was present in 0 (0.0%), while moderate, mild, and little-to-no disability were present in four (10.8%), five (13.5%), and 28 patients (75.7%), respectively.

**Table 4:** Detailed clinical characteristics in patients with headaches.

	***n* = 37**
Unilateral location	23 (62.2)
Pulsating quality	12 (32.4)
Moderate or severe intensity	27 (72.9)
Aggravation by or avoidance of routine physical activity	30 (81.1)
Scintillating scotomas	22 (59.5)
The average duration of headache attack (h)	3 (2–8)
0–10 min	4 (10.8)
10–60 min	3 (8.1)
1–2 h	7 (18.9)
2–4 h	8 (21.6)
4–72 h	15 (40.5)
Monthly numbers of headache days (d)	4 (2–9)
≤ 3 d	12 (32.4)
4–7 d	16 (43.2)
8–14 d	3 (8.1)
15–30 d	6 (16.2)
NRS score	6 (5–8)
≤ 5 (mild)	10 (27.0)
6–7 (moderate)	17 (45.9)
8–10 (severe)	10 (27.0)
HIT−6 score	52 (44–63)
< 49 (little/none impact)	18 (48.6)
50–54 (some impact)	5 (13.5)
55–59 (substantial impact)	1 (2.7)
≥60 (severe impact)	13 (35.1%)
MIDAS score	3 (0–5)
0–5 (little/no disability)	28 (75.7)
6–10 (mild disability)	5 (13.5)
11–20 (moderate disability)	4 (10.8)
≥21 (severe disability)	0 (0.0)

MPNs, myeloproliferative neoplasms; NRS, numerical rating scale; HIT-6, headache impact test-6; MIDAS, migraine disability assessment.

### Management of headaches during the follow-up

Follow-up by face-to-face interview was available for 31 (83.8%) of 37 patients after 6 months. Medications used for acute headaches were NSAIDs in 29 (90.3%) and triptans in nine (29.0%) patients. Headache was managed with low-dose aspirin in 21 (67.7%) patients and with cytoreductive therapy in 17 (54.8%) patients [low-dose aspirin alone: 9 (29.0%); low-dose aspirin combined with cytoreductive therapy: 12 (38.7%); cytoreductive therapy alone: 5 (16.1%)]. Five patients (16.1%) were followed with conservative management (use of NSAIDs or triptans for acute headache) due to mild headache symptoms or lower risk of thromboembolic events.

Table 5 shows a comparison of the detailed characteristics of headaches at baseline and at follow-up. The median number of headache days per month significantly decreased over the time course (baseline: 4 vs. follow-up: 1, *P* < 0.001), as did the average duration of the headache attack (baseline: 3 h vs. follow-up: 2 h, *P* < 0.001). Similarly, the NRS score (baseline: 6 vs. follow-up: 4, *P* < 0.001), HIT-6 score (baseline: 52 vs. follow-up: 36, *P* < 0.001), and MIDAS score (baseline: 2 vs. follow-up: 0, *P* < 0.001) had improved significantly by the follow-up assessment. Laboratory findings, including WBC (*P* = 0.003), Hb (*P* = 0.002), and platelet count (*P* = 0.021) had also decreased by the follow-up interview.

**Table 5:** Comparison of parameters for headache and laboratory findings at baseline and follow-up.

	**Baseline (*n* = 31)**	**Follow-up (*n* = 31)**	***P***
The average duration of headache attack (h)	3 (2–8)	2 (0–3)	<0.001
0–10 min	3 (9.7)	10 (32.3)	
10–60 min	2 (6.5)	4 (12.9)	
1–2 h	7 (22.6)	9 (29.0)	
2–4 h	5 (16.1)	4 (12.9)	
4–72 h	14 (45.2)	4 (12.9)	
Monthly numbers of headache days (d)	4 (2–10)	1 (0–3)	<0.001
≤ 3 d	11 (35.5)	24 (77.4)	
4–7 d	13 (41.9)	5 (16.1)	
8–14 d	1 (3.2)	1 (3.2)	
15–30 d	6 (19.4)	1 (3.2)	
NRS score	6 (5–7)	4 (1–5)	<0.001
≤ 5 (mild)	10 (32.3)	24 (77.4)	
6–7 (moderate)	14 (45.2)	6 (19.4)	
8–10 (severe)	7 (22.6)	1 (3.2)	
HIT-6 score	52 (44–63)	36 (36–40)	<0.001
≤ 49 (little/none impact)	16 (51.6)	29 (93.6)	
50–54 (some impact)	5 (16.1%)	1 (3.2)	
55–59 (substantial impact)	1 (3.2%)	0 (0.0)	
≥60 (severe impact)	9 (24.3%)	1 (3.2)	
MIDAS score	2 (0–4)	0 (0–0)	<0.001
0–5 (little/no disability)	25 (80.6)	30 (96.8)	
6–10 (mild disability)	2 (6.5)	0 (0.0)	
11–20 (moderate disability)	4 (12.9)	1 (3.2)	
≥21 (severe disability)	0 (0.0)	0 (0.0)	
Laboratory findings			
WBC (×10^2^/mm^3^)	85 (70–108)	69 (58–82)	0.003
Hemoglobin (g/dL)	14.0 (13.7–15.2)	13.1 (12.0–14.1)	0.002
Platelets (×10^4^ mm^3^)	71.5 (54.3–81.9)	55.7 (43.4–77.9)	0.021

NRS, numerical rating scale; HIT-6, headache impact test-6; MIDAS, migraine disability assessment; WBC, white blood cell.

**Figure 3:**
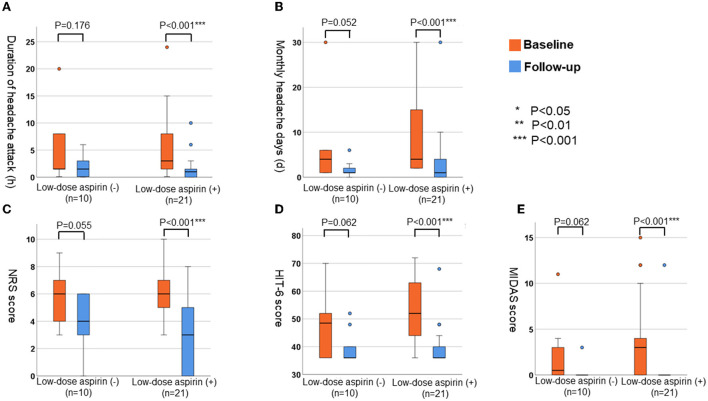
The changes of parameters for headache in patients with or without low-dose aspirin. **(A)** Average duration of headache attack (h). **(B)** Monthly numbers of headache days (d). **(C)** NRS score. **(D)** HIT-6 score. **(E)** MIDAS score.

We compared the parameter for headache at baseline and at follow-up (Figure 3) in patients treated with (*n* = 21) and without (*n* = 10) low-dose aspirin. In patients treated with low-dose aspirin, all variables [average duration of headache attacks, number of headache days per month, NRS score, HIT-6 score, and MIDAS score (all *P* < 0.001)] had significantly improved by the follow-up interview. However, there were no significant differences in these parameters of headache in patients who did not receive low-dose aspirin (*P* = 0.176 for the average duration of headache, *P* = 0.052 for the number of headache days per month, *P* = 0.055 for NRS score, *P* = 0.062 for HIT-6 score, and *P* = 0.062 for MIDAS score).

**Figure 4:**
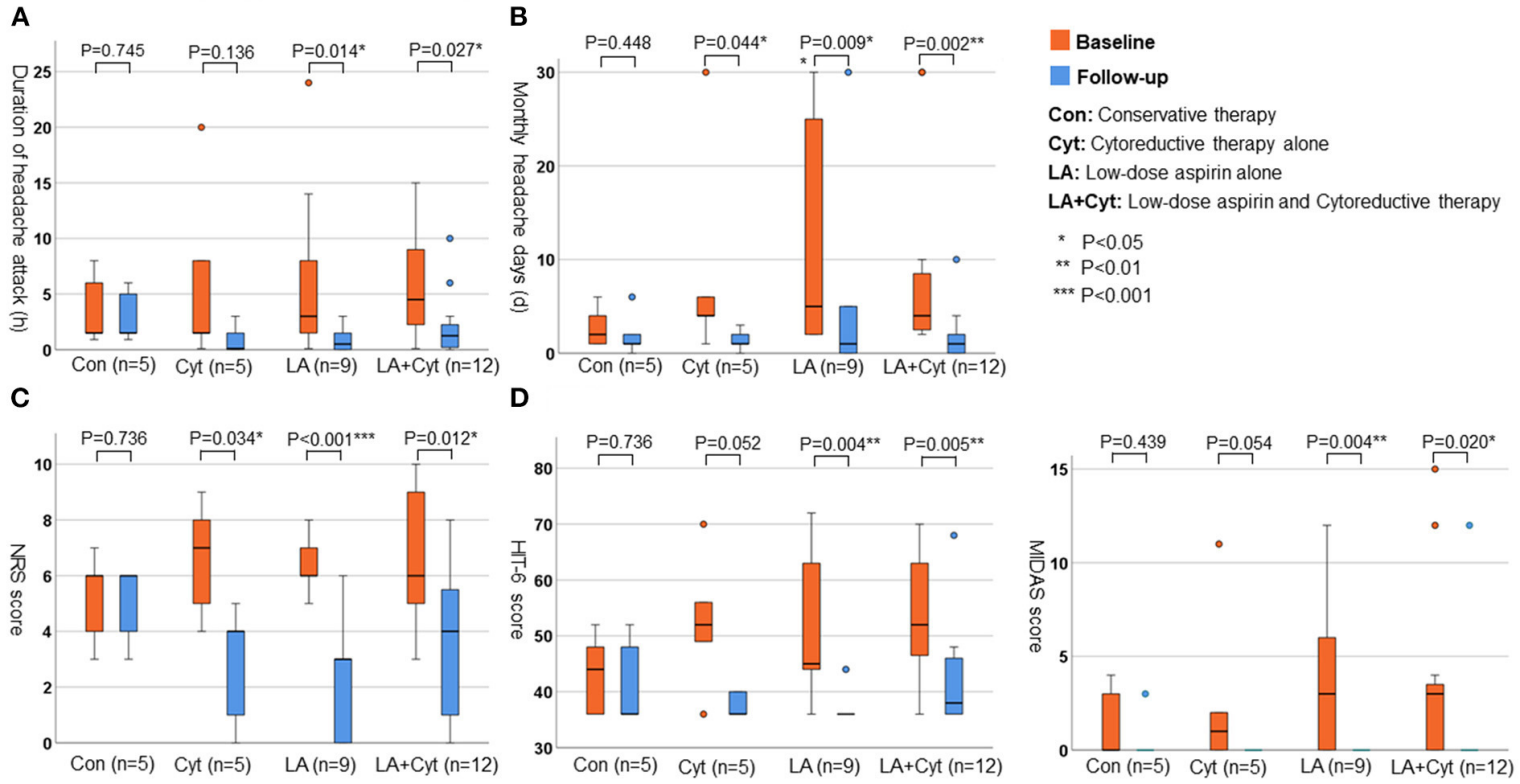
The changes of parameters for headache according to the treatments. **(A)** Average duration of headache attack (h). **(B)** Monthly numbers of headache days (d). **(C)** NRS score. **(D)** HIT-6 score.

Figure 4 indicates the changes in headache parameters according to the management of headaches [conservative therapy (Con; *n* = 5), cytoreductive therapy alone (Cyt; *n* = 5), low-dose aspirin alone (LA; *n* = 9), and low-dose aspirin and cytoreductive therapy (LA+Cyt; *n* = 12)]. In groups treated with low-dose aspirin (LA and LA+Cyt), all variables [average duration of headache attacks (LA: *P* = 0.014; LA+Cyt: *P* < 0.001), number of headache days per month (LA: *P* = 0.009; LA+Cyt: *P* = 0.002), NRS score (LA: *P* < 0.001; LA+Cyt: *P* = 0.012), HIT-6 score (LA: *P* = 0.004; LA+Cyt: *P* = 0.005), and MIDAS score (LA: *P* = 0.004; LA+Cyt: *P* = 0.020)] were significantly improved by the follow-up interview. In contrast, symptoms of headache did not significantly differ in patients with conservative therapy at follow-up (*P* = 0.745 for average duration of attack; *P* = 0.448 for number of headache days per month; *P* = 0.736 for NRS score; *P* = 0.736 for HIT-6 score; and *P* = 0.439 for MIDAS score).

## Discussion

In this prospective observational study, vasomotor symptoms were observed in 50.4% of patients with MPNs. Headache was the most common manifestation of vasomotor symptoms, with a prevalence rate of 27.0% in our cohort. Migraine is a common and often disabling disease in the general population (2). The 2016 Global Burden of Disease data revealed that migraine is the second most disabling condition worldwide, with global age-standardized prevalence of 14.4% overall (2). Vasomotor symptoms have been recognized as transient neurological and ocular manifestations, including headache, paresthesia, visual symptoms, and dizziness, in patients with MPNs (16–19). Previous studies have shown that vasomotor symptoms occur in ~30% of MPN patients (16, 17). Few prospective studies have investigated the prevalence of headaches in patients with MPNs, but reports suggest that between 4 and 39% of these patients experience headache symptoms at some time during the course of the disease (20). One of the few prospective studies noted that five of 37 (13.5%) patients newly presenting with ET complained of headaches (22). Compared to previous reports, the present study had the following strengths: a prospective study design, a larger sample size, genetic analysis of all MPN patients, presentation of clinical details of headache by using the NRS, HIT-6, and MIDAS scores; and assessment of brain MRI. To reach more definitive conclusions regarding the prevalence of headaches in patients with MPNs in comparison with the general population, larger prospective collaborative studies are required.

In the multiple logistic regression analysis, younger age (decrease per 10 years) was the only independent factor associated with headaches in patients with MPNs. Approximately 60% of younger patients (age ≤ 49 years) experienced headaches, and the prevalence rate gradually decreased with advancing age. In contrast, the *JAK2 V617F* mutation and platelet counts were not significantly associated with headaches in patients with MPNs. Although we could not find any significant relationship in the multivariate logistic regression analysis, other variables, including ET, female sex, absence of vascular risk factors, and lack of abnormal MRI findings, were associated with headaches in the univariate analysis. The risks associated with headaches in patients with MPNs have not been well-studied and remain poorly characterized. In general, older age (>60 years), the presence of the *JAK2* V617F mutation, and a history of thrombosis and/or vascular risk factors are considered high-risk features for thrombotic events in patients with MPNs (11–14). Therefore, our findings suggest that factors associated with headaches (e.g., younger age, no vascular risk factors, and lack of abnormal MRI findings) might be opposite to those associated with thromboembolic complications in patients with MPNs. Various mechanisms have been proposed to explain headaches in patients with MPNs. Platelet activation and aggregation, platelet serotonin levels, and platelet nitric oxide levels may play important roles in the incidence of headaches in patients with MPNs (27–29). Michiels et al. (28) reported that shortened platelet survival and an increase in the levels of the platelet activation markers β-thromboglobulin (β-TG), platelet factor 4, and urinary thromboxane B2 were clearly indicative of the spontaneous *in vivo* platelet activation of *JAK2 V617F*-activated thrombocythemic platelets. Interestingly, compared to the controls, β-TG levels were significantly higher in both asymptomatic and symptomatic ET patients (28). Moreover, microemboli and microvascular disturbances may be associated with headaches in patients with MPNs (20, 29), which may produce visual aura attacks by inducing cortical spreading depression (CSD) (30). Indeed, ~60% of patients with headaches had scintillating scotomas in our cohort, which was a higher rate compared to the migraine population (21). Taken together, these interactions, including platelet dysfunction and microvascular disturbance, may predispose MPN patients to focal brain ischemia which can trigger CSD and in turn cause headaches and visual aura.

Headache attacks lasted < 4 h in 22 (59.5%) of 31 patients in the present study. Interestingly, 10% of patients with headaches developed sudden headache attacks that lasted for only 0–10 min. Michels et al. (18) reported “transient cerebral or visual symptoms” related to ET in 17 patients, 10 of whom reported pulsating headache attacks with a sudden onset that lasted a few seconds to several minutes. These findings suggest that some patients with MPNs have headaches with unique characteristics. In this study, about 70% of patients with headaches had moderate to severe intensity headaches (NRS ≥ 6), with 35% of patients suffering a severe impact on life (HIT-6 ≥ 60). The pain intensity as well as the quality of MPN-related headaches (dull, throbbing, or pulsatile) have also been described in previous reports (18–20). However, there have been no reports assessing the detailed clinical features of MPN-related headaches using the NRS, HIT-6, and MIDAS. Regarding visual symptoms including scintillating scotomas, diplopia, and blurred vision in patients with MPNs, all patients underwent brain MRI scanning to assess the presence of stroke or other central nervous system disorders. Moreover, we consulted ophthalmologists to evaluate the secondary cause of visual disturbance if needed. Differential diagnosis from migraine may be difficult, as some MPN-related headache symptoms may be characteristic of conventional migraine attacks. Thus, physicians should perform routine blood tests (e.g., WBC, Hb, and platelet counts) to screen for MPN-related headaches when attending to patients with migraine.

We conducted follow-up by face-to-face interviews with 31 (83.7%) of 37 patients with headaches. Our results indicated that the management of headaches, including low-dose aspirin and/or cytoreduction therapy, was effective in reducing the frequency of headache attacks, pain intensity, and daily disability. In our cohort, ~70% of patients with headaches were treated with low-dose aspirin. All variables (average duration of headache attacks, number of headaches per month, and NRS, HIT-6, and MIDAS scores) were significantly improved by the follow-up interview in these patients. Several reports have shown that low-dose aspirin and reduction of platelet counts to normal levels are useful for the management of MPN-related headaches (18–20, 22–24). Low-dose aspirin relieves peripheral, cerebral, and ocular ischemic disturbances by the irreversible inhibition of platelet cyclooxygenase-1 (COX-1) activity and aggregation (28). In contrast, warfarin, dipyridamole, ticlopidine, sulfinpyrazone, and sodium salicylate have been shown to have no effect on platelet COX-1 activity and were also ineffective in the treatment of vasomotor symptoms, including headaches in patients with MPNs (28). Moreover, some randomized trials, suggested the possibility that daily aspirin in doses from 81 to 325 mg, may be an effective and safe treatment option for the prevention of recurrent migraine headaches (31). Therefore, low-dose aspirin may be considered as the option for the management of MPN-related headaches and migraine. On the other hand, several reports have shown that normalization of platelet counts was also effective in controlling MPN-related headaches (18–20). Although 17 (54.8%) of 31 patients were treated with cytoreductive therapy in this study, these agents were initiated by hematologists based on platelet counts and a higher risk of thrombotic events. Compared with patients treated with cytoreductive therapy alone, combined low-dose aspirin and cytoreductive therapy seemed to be more effective in the management of headaches (see Figure 4). Interestingly, five patients (conservative therapy) were not treated with either low-dose aspirin or cytoreductive therapy. No variables associated with headaches were significantly improved at the follow-up interview in these patients undergoing conservative therapy. At present, low-dose aspirin and/or treatments for MPNs might be the more appropriate management strategies for headaches in patients with MPNs.

The present study had several limitations. First, this was a single-center cohort study conducted in Japan, and the number of patients with headaches was small. Therefore, we could not find a significant relationship of headaches with platelet count and the *JAK2* V617F mutation. Second, ~30% of MPN patients with headaches received aspirin and/or cytoreductive therapy at baseline interview. Third, among 37 patients with headaches, six patients (16.2%) could not be assessed by follow-up face-to-face interview after 6 months, due to death or because they had discontinued follow-up at our hospital. Fourth, headache data (e.g., occurrence, duration, and pain intensity) were collected through participant recall. Finally, the management of headaches and MPNs was conducted based on the attending physician's judgment. Thus, we could not compare the efficacy of low-dose aspirin compared to the placebo group. The presence of recall bias for the collection of headache data and selection bias for treatment strategies may have had potentially significant confounding effects on the average duration of headache attacks, the monthly number of headaches, and NRS, HIT-6, and MIDAS scores at the follow-up interview. Large prospective collaborative studies are required to reach more definitive conclusions regarding the characteristics of MPN-related headaches and their management.

## Conclusion

Headache was the most common vasomotor symptom in patients with MPNs. Younger age, but not platelet counts or the *JAK2V 617F* mutation, were independently associated with headaches in patients with MPNs. In some patients, headache may have a sudden onset and brief duration lasting only 0–10 min. Most MPN-related headaches may be managed with low-dose aspirin and MPN control.

## Data availability statement

The raw data supporting the conclusions of this article will be made available by the authors, without undue reservation.

## Ethics statement

The studies involving human participants were reviewed and approved by Nippon Medical School. The patients/participants provided their written informed consent to participate in this study.

## Author contributions

TS, FS, TH, SW, HY, and KK made substantial contributions to conception and design, acquisition of data, and analysis and interpretation of data. TS, HY, and KK have been involved in drafting the manuscript or revising it critically for important intellectual content. All authors participated sufficiently in the work to take public responsibility for appropriate portions of the content and agreed to be accountable for all aspects of the work in ensuring that questions related to the accuracy or integrity of any part of the work are appropriately investigated and resolved. All authors have read and approved the manuscript.

## Conflict of interest

The authors declare that the research was conducted in the absence of any commercial or financial relationships that could be construed as a potential conflict of interest.

## Publisher's note

All claims expressed in this article are solely those of the authors and do not necessarily represent those of their affiliated organizations, or those of the publisher, the editors and the reviewers. Any product that may be evaluated in this article, or claim that may be made by its manufacturer, is not guaranteed or endorsed by the publisher.
